# CD15s/CD62E Interaction Mediates the Adhesion of Non-Small Cell Lung Cancer Cells on Brain Endothelial Cells: Implications for Cerebral Metastasis

**DOI:** 10.3390/ijms18071474

**Published:** 2017-07-10

**Authors:** Samah A. Jassam, Zaynah Maherally, James R. Smith, Keyoumars Ashkan, Federico Roncaroli, Helen L. Fillmore, Geoffrey J. Pilkington

**Affiliations:** 1 Brain Tumour Research Centre, Institute of Biomedical and Biomolecular Sciences, School of Pharmacy and Biomedical Sciences, University of Portsmouth, Portsmouth PO1 2DT, UK; samah.jassam@port.ac.uk (S.A.J.); zaynah.maherally@port.ac.uk (Z.M.); james.smith@port.ac.uk (J.R.S.); helen.fillmore@port.ac.uk (H.L.F.); 2 Neuro-Surgery, King’s College Hospital, Denmark Hill, London SE5 9RS, UK; k.ashkan@nhs.net; 3 Division of Neuroscience and Experimental Psychology, Faculty of Biology, Medicine and Health, Oxford Road, Manchester M13 9PT, UK; federico.roncaroli@manchester.ac.uk

**Keywords:** CD15s, Sialyl Lewis X, CD62E, brain metastasis, adhesion

## Abstract

Expression of the cell adhesion molecule (CAM), Sialyl Lewis X (CD15s) correlates with cancer metastasis, while expression of E-selectin (CD62E) is stimulated by TNF-α. CD15s/CD62E interaction plays a key role in the homing process of circulating leukocytes. We investigated the heterophilic interaction of CD15s and CD62E in brain metastasis-related cancer cell adhesion. CD15s and CD62E were characterised in human brain endothelium (hCMEC/D3), primary non-small cell lung cancer (NSCLC) (COR-L105 and A549) and metastatic NSCLC (SEBTA-001 and NCI-H1299) using immunocytochemistry, Western blotting, flow cytometry and immunohistochemistry in human brain tissue sections. TNF-α (25 pg/mL) stimulated extracellular expression of CD62E while adhesion assays, under both static and physiological flow live-cell conditions, explored the effect of CD15s-mAb immunoblocking on adhesion of cancer cell–brain endothelium. CD15s was faintly expressed on hCMEC/D3, while high levels were observed on primary NSCLC cells with expression highest on metastatic NSCLC cells (*p* < 0.001). CD62E was highly expressed on hCMEC/D3 cells activated with TNF-α, with lower levels on primary and metastatic NSCLC cells. CD15s and CD62E were expressed on lung metastatic brain biopsies. CD15s/CD62E interaction was localised at adhesion sites of cancer cell–brain endothelium. CD15s immunoblocking significantly decreased cancer cell adhesion to brain endothelium under static and shear stress conditions (*p* < 0.001), highlighting the role of CD15s–CD62E interaction in brain metastasis.

## 1. Introduction

The process of cancer metastasis from one organ to another involves many stages [1,2]. However, the mechanisms by which cancer cells adhere to and cross the neurovascular unit and subsequently colonise the brain tissue are not fully substantiated. CD15s (Sialyl Lewis X) is known to be a marker associated with neoplastic cells and is a natural E-selectin (CD62E) ligand [3,4]. Some cancer cells use a similar mechanism to that used by extravasating white blood cells (WBCs) whereby cell surface CD15s binds with endothelial cell CD62E. This CD15s expression and its role in binding to the brain vascular endothelium via CD62E may explain why some cancer cells have greater brain metastatic potential than others. The molecular mechanisms underlying the complex communication between cancer cells and brain endothelium during brain metastasis, however, require further elucidation.

### 1.1. The Role of CD15s in Cancer Metastasis

The tetra-polysaccharide molecule CD15s (SLe^x^), expressed on the terminus of cell surface glycolipids, acts as a ligand for the selectins CD62E (E-selectin) and CD62P (P-selectin) [5], being centrally involved with neoplastic cell to vascular endothelial cell adhesion [5,6,7,8,9]. The homing process of circulating WBCs through tethering and rolling is initially facilitated via CD15s/CD62E heterophilic binding at injury sites [10,11]. Moreover, metastatic colon adenocarcinoma cells have also been shown to adhere to human umbilical vein endothelial cell (HUVEC) through CD15s/CD62E interaction [9]. An additional link between CD15s and malignancy was made on both human non-small cell lung cancer (NSCLC) and human primary liver cancer (PLC) in tissue sections [12] while CD15s has also been reported to be expressed on 51% of advanced primary gastric cancer and metastatic lymph nodes and on 60% of primary gastric cancer patients [13]. CD15s has also been utilized as an early diagnostic maker for cervical cancer [14]. The expression of CD15s in brain metastases has, however, hitherto been under-investigated. Having recently reported CD15s overexpression to be correlated with cell cycle arrest at G1 phase in both glioblastoma (GBM) cells and brain metastatic cells from lung cancer [15], we sought to investigate its possible involvement in the initial stages of brain metastasis.

### 1.2. Role of CD62E (E-Selectin) in Cancer Metastasis

The glycoprotein CD62E, a cell adhesion molecule (CAM), expressed on brain endothelium [16] following stimulation with tumour necrosis factor alpha (TNF-α) and other cytokines [16,17] can bind fucosylated and sialylated CD15 on WBCs and neoplastic cells [18,19]. A high affinity between CD62E and CD15s has also been recognised though crystallographic investigation [19]. Other CD62E ligands include CD44, CD107a and CD107b [20,21,22]. In addition, in vivo and in vitro studies have shown that interactions between CD62P/CD62E and various glycoconjugates mediate the WBC tethering process during inflammation [23] while the role of CD62E/CD15s interaction in endothelial binding of circulating cancer cells (CCCs) during the metastatic cascade has been suggested [24]. Indeed, endothelial cell overexpression of CD62E was reported in metastatic lung cancer foci [25] while in spontaneous murine astrocytoma (SMA-560) model systems transendothelial migration was reduced by monoclonal antibody blocking of CD62E [26]. Considering the clinical importance of brain metastasis and the therapeutic challenges, it is crucial to find a way to prevent NSCLC cells from crossing the blood–brain barrier (BBB). Thus, this study aims to develop our understanding of the molecular mechanisms underlying metastasis from NSCLC to brain, specifically focussing on the adhesion of NSCLC cells to brain-derived endothelial cells which compose an important part of the neuro-vascular unit and constitute the first major obstacle to colonization of the brain by metastatic cancer cells.

## 2. Results

### 2.1. Characterisation of CD15s in Cultured Cells

**Figure 1:**
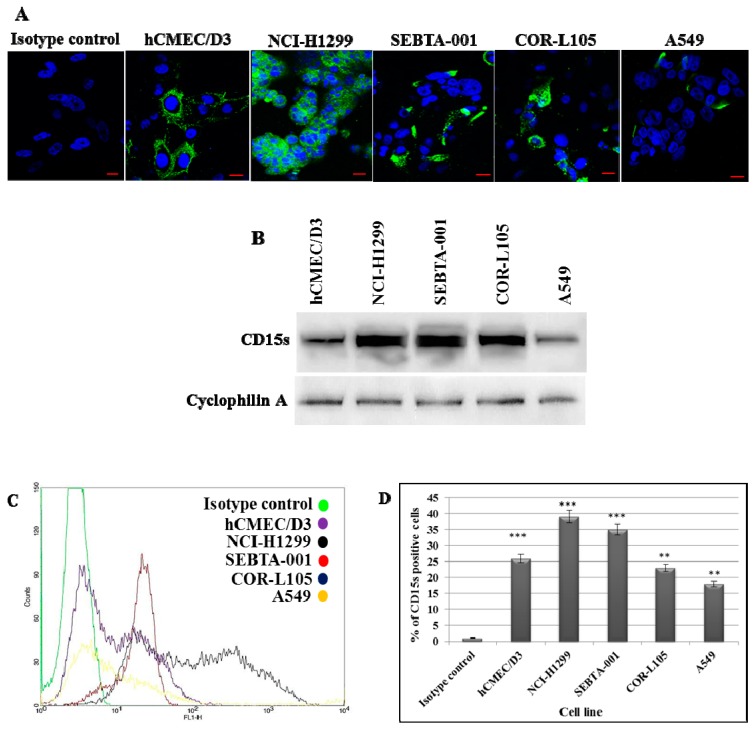
Expression of CD15s in brain endothelial and lung cancer cell lines: (**A**) Representative ICC images showing CD15s in hCMEC/D3, NCI-H1299, SEBTA-001, A549 and COR-L105. Hoechst blue was used as a nuclear counterstain. Scale bar = 20 µm; (**B**) Western blot analysis of CD15s of proteins from the cell lines showed highest CD15s expression in NCI-H1299 and SEBTA-001 followed by hCMEC/D3, COR-L105 and A549. Cyclophilin A was used as a protein loading control; (**C**) representative flow cytometric histograms (Green: control, purple: hCMEC/D3, black: NCI-H1299, red: SEBTA-001, blue COR-L105 and yellow: A549); (**D**) quantitative flow cytometric analysis of CD15s expression on hCMEC/D3, NCI-H1299, SEBTA-001, COR-L105 and A549. CD15s was overexpressed in NCI-H1299 and SEBTA-001 with less expression in hCMEC/D3, COR-L105 and A549. *n* = 3, *** *p* < 0.001 and ** *p* < 0.01. Data is expressed as ±SE.

Localisation and extracellular expression of CD15s were characterised in human brain endothelial cells as well as primary and metastatic NSCLC cells via immunocytochemistry (ICC), Western blotting (WB) and flow cytometry (FC) analysis. CD15s was localised and prominently expressed on the surface of brain metastatic lung cancer cells (SEBTA-001 and NCI-H1299). Lower expression levels were seen on the surface of brain endothelial cells (hCMEC/D3) and on primary lung cancer cell lines (COR-L105 and A549) compared with the isotype control (Figure 1A). Semi-qualitative WB analysis showed intense immunopositive bands in the metastatic cells while less intensity was observed in primary cancer cells and endothelial cell membrane extracts (Figure 1B). Qualitative FC analysis showed highest CD15s positivity was observed in metastatic lung cancer cells, NCI-H1299 (39%) and SEBTA-001 (35%), followed by brain endothelial cells (hCMEC/D3) (25%) and primary lung cancer cell lines (COR-L105 (21%) and A549 (18%)) (Figure 1C,D). Note that, while Western blot data suggest COR-L105 has high CD15s expression, ICC and FC results did not correlate, showing relatively low expression of this epitope. This probably reflects the different antibodies used in the WB and ICC/FC, as well as the techniques to quantity the expression level.

### 2.2. The Absence of CD62E Reduced Cancer Cell–Brain Endothelium Adhesion

**Figure 2:**
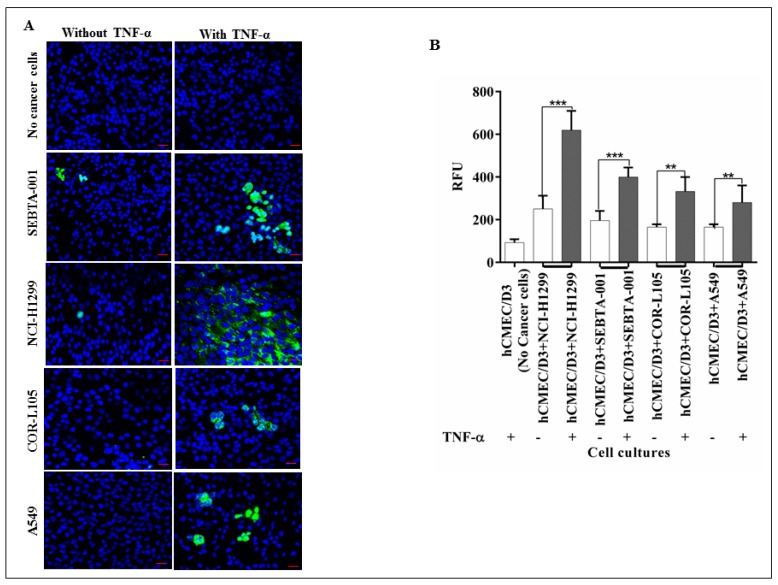
The role of CD62E in adhesion of NSCLC cells to brain endothelium: (**A**) Qualitative adhesion of NSCLC cells onto brain endothelium monolayer. Green fluorescently tagged NSCLC cells were applied onto the hCMEC/D3 monolayer and incubated for 90 min with and without activation via TNF-α. Non-adherent cells were washed and co-cultures were fixed and examined by confocal microscopy; (**B**) quantitative adhesion of NSCLC cells. hCMEC/D3 cells were seeded into 96-well plate followed by seeding of green fluorescently tagged NSCLC cells on the hCMEC/D3 monolayer and incubated for 90 min incubation. Non-adherent cells were washed out and adherent cells were lysed followed by quantification via a microplate reader at 480–520 nm. Results showed a strong decrease in adhesion caused by absence of TNF-α (White bar) compared to TNF-α stimuli. *n* = 3, *** *p* < 0.001 and ** *p* < 0.01. Scale bar = 20 µm. Data is expressed as ±SE.

To explore the role of CD62E in adhesion of NSCLC cell to brain endothelium, we conducted qualitative and quantitative adhesion assays under static conditions. CD62E expression was first activated by TNF-α (25 pg/mL). Green fluorescently tagged NSCLC cells were then applied onto activated and non-activated brain endothelial cells. Findings showed that absence of CD62E significantly reduced the adhesion of all cancer cells (*p* < 0.001) (Figure 2) compared to the high numbers of adherent cells on activated brain endothelial cells expressing CD62E. These results suggest that CD62E and TNF-α have a key role in adhesion of NSCLC during seeding into the brain.

### 2.3. Immunoblocking of CD15s Reduced Adhesion of Cancer Cell–Brain Endothelium under Static Conditions

**Figure 3:**
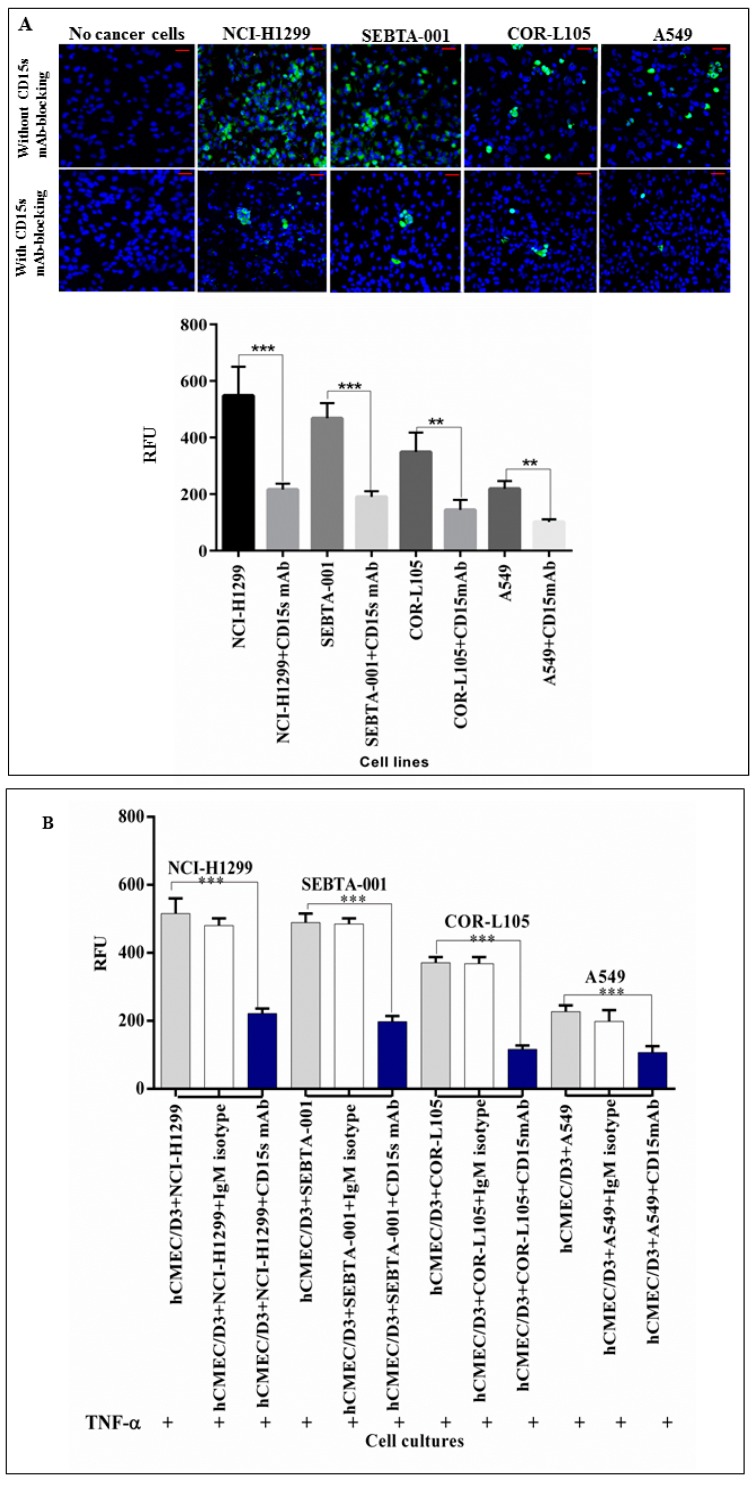
(**A**) CD15s immunoblocking reduced the adhesion of lung cancer cells under static conditions. Confocal images (top panel) showing adhesion of green fluorescently labelled NSCLCs on a brain endothelial cell monolayer (blue) and semi-quantitative analysis of confocal images (lower panel) showed a significant decrease in adhesion ability of NSCLC cells to adhere to hCMEC/D3 cells. *n* = 3, *** *p* < 0.001 and ** *p* < 0.01. Scale bar = 20 µm. Data is expressed as ±SE. We used the same concentration of TNF-α as optimised in a previous study [5] to activate the expression of CD62E on brain endothelial cells. Brain endothelial cells (hCMEC/D3) were exposed to 25 pg/mL of TNF-α for 18 h and levels of CD62E were detected using flow cytometry (Data not shown); (**B**) blocking with CD15s mAb significantly decreased the adhesion of NSCLC to brain endothelium. Quantitative adhesion assay of human primary and metastatic lung cancer (NSCLC) cells blocked with CD15s mAb (blue bar), non-specific isotype IgM (white bar) to assess the efficiency and specificity of CD15s mAb-blocking efficiency and NSCLC cells on a monolayer of hCMEC/D3 (grey bar) as a negative control. *n* = 3, *** *p* < 0.001. Data is expressed as ±SE.

A qualitative adhesion assay under static conditions was performed using a confocal microscope and quantitatively using a plate reader to assess the role of CD15s in adhesion. Results showed that metastatic cancer cells (NCI-H1299 and SEBTA-001) were more adherent than primary lung cancer cell lines (COR-L105 and A549) (Figure 3). Immunoblocking of CD15s significantly (*p* < 0.001) reduced adhesion of cancer cells onto an activated brain endothelial cell monolayer. These results suggested a correlation between the expression of CD15s and endothelial cell adhesion of lung cancer cells (Figure 3A). In addition, mAb-immunoblocking against CD15s reduced the adhesion of cancer cells compared to the adhesion ability of cancer cells without mAb-CD15s immunoblocking. However, no decrease in adhesion was detected during blocking with non-specific isotype (IgM) monoclonal antibodies. These results confirmed the specificity of mAb-CD15s blocking and validated the correlation of CD15s and adhesion ability of cancer cells under static conditions (Figure 3B).

### 2.4. CD15s mAb Blocking Decreases Adhesion of NSCLC Cells under Shear Stress Condition

**Figure 4:**
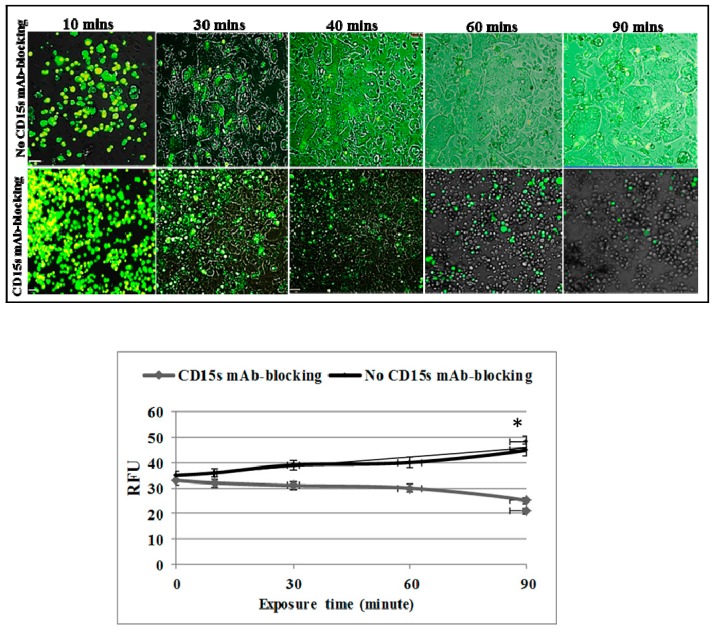
Immunoblocking with CD15s mAb significantly decreased the adhesion ability of NSCLC under dynamic conditions (2.5 dyn/cm^2^). A dynamic cell adhesion assay was carried out on highly metastatic brain cells (SEBTA-001) using an AxioVert 200 M microscope. (**Top panel**) Cancer cells were incubated with isotype control (IgM) or CD15 mAb followed by perfusion of 1 × 10^6^ cancer cells over a monolayer of hCMEC/D3 cells at 2.5 dyn/cm^2^ for 90 min. Phase contrast and fluorescent images were acquired at real time every 10 min with a ×5 objective using Volocity software. Scale bar = 20 µm. (**Lower panel**) Results are shown in relative fluorescent units of adherent cancer cells with and without CD15 mAb for 10, 20, 30, 40, 60 and 90 min time points. * *p* < 0.05. Data is expressed as ±SE.

To determine whether CD15s plays a role in adhesion of cancer cells under physiological shear stress (blood flow conditions), an activated endothelial monolayer was allowed to grow on a Vena8 endothelial+ biochip and green fluorescent tagged NSCLC cells were perfused onto the endothelial monolayer via a micropump (Cellix, Dublin, Ireland). Live cell microscopy was then conducted to monitor the effect of CD15s immunoblocking on the adhesion of cancer cells. A highly metastatic lung to brain cancer cell line (SEBTA-001) was perfused at a rate of 2.5 dyn/cm^2^ with pre-warmed fresh EBM + EGM2 medium supplemented with 2% human serum and 25 pg/mL TNF-α. Cell adhesion was then studied over a 90-min time range. Results showed that SEBTA-001 cells adhered onto the activated endothelial monolayer where no CD15s immunoblocking was applied. The number of adherent cells was also seen to increase in a time dependent manner (Figure 4). In parallel, the same number of SEBTA-001 cells (previously incubated with mAb-CD15s for 10 min) was perfused onto the activated endothelial monolayer and no adhesion was seen. The cancer cells stayed in suspension (Figure 4 and supplementary materials). These findings confirmed the key role of CD15s in adhesion of lung cancer cells to brain endothelial cells under physiological shear stress conditions.

### 2.5. CD15s Expression Was Localised at the Peripheral Adhesion Sites of Cancer Cell–Brain Endothelium

**Figure 5:**
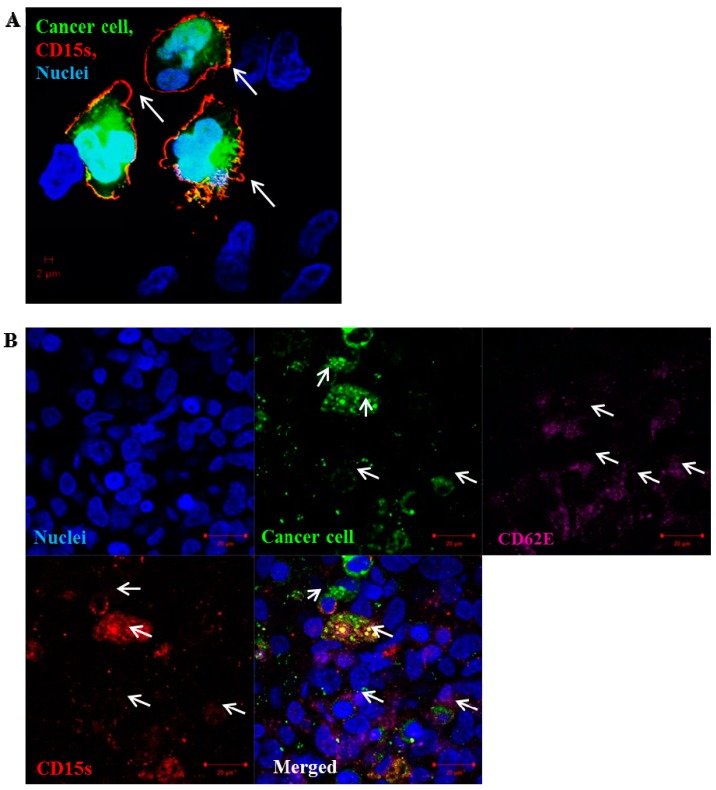
Localisation of CD15s on the surface of adherent SEBTA-001 cells at the site of adhesion. Confocal image of green fluorescently-labelled adherent brain to lung metastatic cancer cells (SEBTA-001) on a monolayer of activated hCMEC/D3 cells (blue). (**A**) Expression of CD15s (red) (white arrows) was seen on the edges of adherent cells SEBTA-001 (green) at the site of adhesion on brain endothelial cells; (**B**) expression of CD15s (red) (white arrows) was observed on surface of adherent cancer cells SEBTA-001 (green) and CD62E (purple) (white arrows) was seen on the monolayer of activated brain endothelial cells (hCMEC/D3) at adhesion site of cancer cell–brain endothelium. Scale bar = 20 µm.

Results showed a prominent expression of CD15s (red) on the surface of green fluorescently tagged cancer cells with accumulation on the surrounding outer edges and processes of the adherent cancer cells (Figure 5A). CD15s and CD62E were both co-localised at the site of NSCLC cell adhesion where CD15s (red) was seen to be expressed on green-tagged cancer cells and CD62E (purple) observed on the surface of the activated brain endothelial cells at the site of the adhesion (Figure 5B). These images assessed the involvement of CD15s–CD62E interaction in adhesion process of NSCLC cells onto brain endothelium.

### 2.6. CD15s Expression in Human Biopsy of Lung to Brain Metastasis

**Figure 6:**
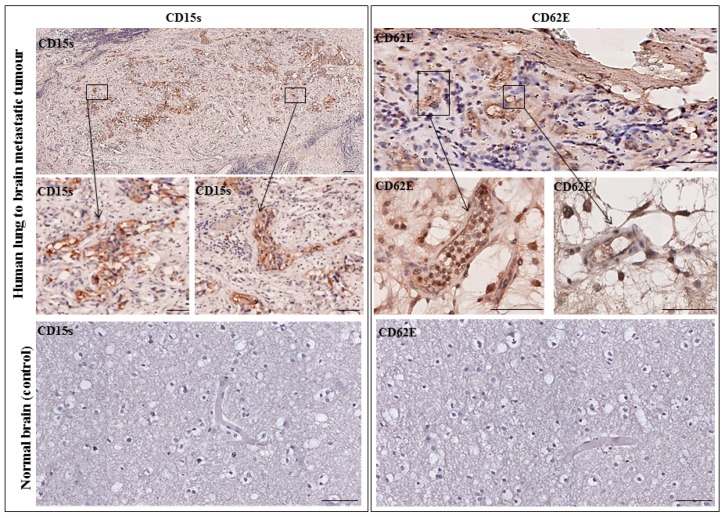
Representative immunohistochemistry images from one patient with lung–brain metastasis. (**Left panel**) CD15s was detected in tumour core and infiltrated into non-neoplastic host brain tissue. No expression was seen in a normal brain section. (**Right panel**) CD62E was expressed on the inner lining of brain endothelial cells with no expression seen in normal brain sections. Images were obtained using an Ariol microscope (Leica, Wetzlar, Germany) at 20× and 40× magnification. Scale bar = 20 µm. *n* = 1.

Paraffin embedded formalin fixed tissue sections of human lung to brain metastatic tumour biopsy and normal brain tissue sections were immunostained for CD15s and CD62E. Results showed that CD15s was expressed in brain metastatic tumour sections. CD15s positive cells were seen throughout the tumour core and in the distant local lesions. An area of host tumour interface was seen where CD15s positive cells were detected within vessels. No CD15s expression was observed in the non-malignant adjacent host brain tissue and in normal brain tissue section (Figure 6, left panel). In parallel, CD62E was expressed on the inner lining of brain endothelial cells with no expression seen in normal brain sections (Figure 6, right panel). These results highlight the importance of CD15s and CD62E in brain metastasis.

## 3. Discussion

Brain metastasis is a complicated, multi-step process, the underlying molecular mechanisms of which are still not fully understood. Twenty to forty per cent of non-small cell lung cancer (NSCLC) patients develop brain metastasis at or within a short period of primary tumour diagnosis [27]. Metastasis to the brain requires efficient biological interaction between the metastatic cells and their vascular microenvironment. Circulating cancer cells have to go through tethering, rolling, adhesion and transmigration during seeding into the brain. Little, however, is known about the molecular basis of these interactions, particularly, the adhesion process between cancer cell and brain vascular endothelium. CD15s is a tetrasaccharide cell–cell adhesion and cell recognition molecule [28], which is involved in adhesion of lymphocyte during the homing process [29]. CD15s overexpression was reported to correlate with distant metastasis in lung cancer patients [30], as well as in colorectal metastatic cancer [31] and liver metastasis where CD15s inaction with selectins mediates adhesion of cancer cells onto liver endothelium [32]. In addition, elevated levels of CD15s were revealed to be associated with metastasis in head and neck cancer [33]. Our study showed that CD15s was expressed on the cell surface of brain endothelial cells (hCMEC/D3), primary NSCLC cell lines (A549 and COR-L105) while the highest levels of expression were observed on NCI-H1299, derived from a metastatic NSCLC from cervical lymph node, and SEBTA-001, a metastatic NSCLC obtained from the brain (Figure 1). These results correlated with the findings of Fukuoka et al. (1998) [30] who revealed that high CD15s expression was correlated with metastasis in lung carcinoma. Similar findings were shown by Kadota et al. (1999) [34], suggesting CD15s correlates with distant metastatic lesions in lung carcinoma. Recently, elevated levels of CD15s were reported to be used as an independent marker to predict NSCLC patients at the metastatic stage [35], although, little is known about CD15s expression in CNS malignancies. We recently reported that CD15s overexpression was cell cycle dependent in high grade glioblastoma cells as well as in metastatic brain tumour cells originating from lung [36]. It has also been shown that brain endothelial cells play a key role in metastasis to the brain [37]. An adult human brain microvascular endothelial cell line (hCMEC/D3) was used in our studies [38]. This brain derived endothelial cell line has been well characterised in an all human in vitro BBB model [15,39,40]. We previously optimised the concentration of TNF-α (25 pg/mL) needed to trigger the extracellular expression of CD62E on hCMEC/D3 cells [36]. All studied cells were maintained in medium supplemented with human serum to avoid any possible functional changes enhanced by bovine serum [41,42]. Previously, non-CNS endothelial cells such as HUVECs have been used to study metastasis to the brain [43]. A functional difference however was subsequently noted in adhesion of leukocytes on brain endothelial cells and adhesion on umbilical vein endothelial cells [44]. The use of all human, brain-derived cellular models is, therefore, of paramount importance [5,39]. The importance of CD15s in cell–cell adhesion is due to its propensity to interact with endothelial adhesion molecules such as E-selectin (CD62E), as in the lymphocyte homing process [45,46]. Interestingly, our findings showed a correlation between expression of CD15s and adhesion. Less adhesion was seen in primary lung cancer cells (COR-L105 and A549) compared to highly metastatic cells (NCI-H1299, SEBTA-01 and SEBTA-005) (Figure 2). A significant decrease in NSCLC adhesion was observed by the absence of CD62E and in turn without TNF-α stimuli (Figure 2). Earlier studies showed the involvement of CD15s–CD62E interaction in metastasis in different non-CNS cancers and revealed that entrapping the interaction between CD15s and CD62E largely reduced the adhesion of lung carcinoma, colon cancer and macrophages to endothelial cells [47]. We found that a significant decrease (*p* < 0.01) in adhesion of lung cancer cells was caused by blocking with CD15s monoclonal antibodies (Figure 3). These results suggest that both CD15s and CD62E play an important role in adhesion of cancer cell onto brain endothelium under static conditions. We then investigated whether blocking of CD15s reduces the adhesion of cancer cells under a physiological flow system. We conducted live cell imaging of cell–cell adhesion under shear stress at a perfusion rate of 2.5 dyn/cm^2^ and volumetric flow rate of 10 mL/h resembling that of the blood flow in the human brain microvascular system [48]. Using metastatic NSCLC cells obtained from brain (SEBTA-001), results showed that blocking with antibodies against CD15s reduced cell adhesion compared with non-blocked cells (Figure 4). Confocal images revealed CD15s immunopositive cells surrounding the adherent cells and co-localisation of CD15s/CD62E was seen at the site of adhesion of metastatic lung cancer cells (SEBTA-001) onto brain endothelial cells (Figure 5). These findings suggest CD15s and CD62E play a key role in lung cancer cell adhesion. CD15s was also seen to be expressed in human tissue sections of lung to brain metastasis while, no CD15s was detected in the normal brain tissue sections (Figure 6). Although little is known regarding the in vivo and in vitro characterisation of CD15s expression in the human CNS, a single study revealed that CD15s was not expressed in any type of normal brain cell apart from microglia [49].

Results from this present study, wherein a specialised in vitro experimental model was used to investigate cell–cell adhesion of metastatic lung cancer cells to brain endothelial cells provide supporting the role of CD15s/CD62E interactions in lung to brain metastasis and may serve as a potential therapeutic target. CD15s and its potential role in the binding of circulating tumour cells to the brain endothelial cells is quite significant given the fact that this may be a step in the multi-step metastasis process that could be targetable. Preventing the circulating tumour cells from the initial binding step offers a novel approach to prevention of brain metastasis in patients diagnosed with NSCLC.

## 4. Materials and methods

### 4.1. Ethics Statement

All cell lines established “in house” were conducted in accordance with the National Research Ethics Service (NRES) instructions and under Ethics permission 11/SC/0048 (received: 29 August 2016).

### 4.2. Cell Culture

The human cerebral microvascular endothelial cell line (hCMEC/D3) was donated by Pierre Olivier Couraud (Institute of Cochin, INSERM, Paris, France) [50]. These cells were cultured in endothelial basal medium-2 (EBM-2; Lonza, Slough, UK) supplemented with Vascular Endothelial Growth Factor (VEGF), human Epidermal Growth Factor (hEGF), R3-Insulin-like Growth Factor-1 (R3-IGF-1), Ascorbic Acid, Hydrocortisone, human Fibroblast Growth Factor-Beta (hFGF-β), Heparin (Lonza, Slough, UK) and 2% human serum (Sigma-Aldrich, Gillingham, UK). Primary NSCLC cell lines (A549 and COR-L105) were purchased from Sigma-Aldrich (Gillingham, UK); metastatic NSCLC from cervical lymph nodes (NCI-H1299) from ATCC^®^ and low passage brain-metastatic NSCLC (SEBTA-001) was established “in house” from biopsies derived from patients with lung–brain secondary tumours. All NSCLC cells were cultured in Dulbecco’s Modified Eagle Medium supplemented with 2% human serum (Sigma-Aldrich, Gillingham, UK) and maintained at 5% CO_2_ and a humidified atmosphere at 37 °C. All cell lines were routinely tested for mycoplasma using the MycoAlert™ (Lonza, Slough, UK) kit. Cell authentication was also carried out routinely using a microfluidic electrophoresis system via an Agilent 2100 Bioanalyzer (Agilent Technologies, Santa Clara, CA, USA), to analyse STR-PCR fragments from 10 human genomic loci of human cell lines [50].

### 4.3. Antibodies

#### 4.3.1. Primary Antibodies

Mouse monoclonal anti-CD15s (KM93) (Millipore, ThermoFisher, East Grinstead, UK) was used at the following dilutions based on the company’s recommended working range: 1:50 for immunocytochemistry (ICC), 1:50 for Western blot (WB), 1:10 for flow cytometry (FC) and immuno-blocking and 1:100 for immunohistochemistry (IHC). Mouse monoclonal anti-CD62E (Sigma-Aldrich, Gillingham, UK) was used at the following dilutions: 1:100 for ICC, 1:25 for FC, 1:200 for WB and 1:200 for IHC. Rabbit polyclonal anti-ABCE1 (Novus Technologies, Singapore, Singapore) was used as a loading control for WB at 1:500.

#### 4.3.2. Secondary Antibodies

Fluorochrome-conjugated Alexa Fluor-488, Alexa Fluor-568 and Alexa Fluor-647 (Invitrogen, Carlsbad, CA, USA) was used for ICC and FC at 1:500. Horseradish peroxidase (HRP)-conjugated IgG (Invitrogen, Carlsbad, CA, USA) was used at 1:2500 for chemiluminescent detection in WB.

#### 4.3.3. Isotype CONTROLS

To confirm the specificity of primary antibody binding, isotype control antibodies were used (IgG and IgM) (Invitrogen, Carlsbad, CA, USA) at 1:100 for ICC, 1:10 for FC and 1:10 for immunoblocking for CD15s. IgG Isotype control antibodies (Invitrogen) at dilutions 1:50 for ICC and 1:10 for flow cytometry for CD62E controls.

### 4.4. Immunocytochemistry (ICC)

Cells were seeded onto sterile coverslips at 1 × 10^5^/well and left to incubate overnight at 5% CO_2_ and a humidified atmosphere at 37 °C. Cells were fixed with 4% Paraformaldehyde (PFA) (Sigma-Aldrich, Gillingham, UK) followed by three washes with Phosphate buffered saline (PBS) (Sigma-Aldrich, Gillingham, UK). Cells were then blocked with 10% goat serum (Sigma-Aldrich, Gillingham, UK), incubated with the primary antibody overnight followed by incubation with secondary antibody for 45 min. Cells were then counterstained with 1 mg/mL of Hoechst Blue (Sigma-Aldrich, Gillingham, UK). Coverslips were mounted on slides using Vectashield^®^ (Vector Laboratories, Burlingame, CA, USA) and viewed using a Zeiss Axio Imager Z1 fluorescence microscope. Images were captured using Volocity Image Analysis Software (V 5.2, PerkinElmer, Waltham, MA, USA).

### 4.5. Flow Cytometry (FC)

Cells were harvested by gentle scraping, blocked with 2% goat serum and probed with primary antibody for 30 min at 4 °C. Isotype antibodies were added to the control wells to ensure specificity of the primary antibodies. Cells were then washed twice with PBS and incubated with secondary antibody for 20 min at 4 °C. Cells were re-suspended in PBS then transferred to Fluorescence-activated cell sorting (FACS) tubes (BD Biosciences, BD Biosciences, Oxford, UK). Propidium iodide (PI) (Sigma-Aldrich, Gillingham, UK) was added to samples for extracellular antigen detection. Analysis was performed using a four-colour multi-parameter FACS Calibur (BD Biosciences, Oxford, UK) machine. Each experiment was repeated independently three times in triplicate. The expression level was assessed by percentage of positive cell population.

### 4.6. Western Blotting (WB)

Cells were lysed using RIPA buffer (ThermoFisher, East Grinstead, UK) with 1× Halt Protease Phosphatase Inhibitor Cocktail (ThermoFisher, East Grinstead, UK). Lysate protein concentration was determined by the Pierce BCA Protein assay (ThermoFisher, East Grinstead, UK). Lysates were separated by SDS-PAGE and protein loaded onto 1.5 mm thick 10% SDS-Polyacrylamide gels and run for 90 min at 100 V. Proteins were then transferred to methanol-activated Immun-Blot PVDF membranes (BioRad, Watford, UK) using the Pierce Power System (ThermoFisher, East Grinstead, UK) (10 min; 10 Volt; 1.2 Ampere). Membranes were then blocked with 5% milk buffer (Marvel, UK) for 1 h at room temperature (RT). Incubation with the primary antibody was performed overnight at 4 °C. Membranes were washed 3 times with TBST before staining with the secondary antibody conjugated to a NIR-dye for 1 h at RT. Prior to scanning, the membranes were washed 3 times with TBST and once with TBS. Blots were scanned on a Li-cor Odyssey CLx and analysed using ImageStudio V5.2 (Licor, Lincoln, NE, USA).

### 4.7. Adhesion Assays

#### 4.7.1. Quantitative Adhesion Assay

Adhesion ability of tumour cell–brain endothelial cell was evaluated using the CytoSelect™ Tumor-Endothelium Adhesion Assay Kit (Cell Biolabs, San Diego, CA, USA). Briefly, 48-well plate (Corning, Corning, NY, USA) was coated with 10 µg/mL of fibronectin (Sigma-Aldrich, Gillingham, UK). Then, 1 × 10^6^ cells/well hCMEC/D3 were seeded and grown to 80% confluency. To activate the extracellular expression of CD62E, hCMEC/D3 cells were incubated at 25 pg/mL of TNF-α. In parallel, NSCLC cells were stained with live cell green fluorescent dye (Cyto Tracker™) (Cell Biolabs, San Diego, CA, USA) for 1 h. Then, 1 × 10^5^ of the stained NSCLC cells were seeded onto the activated hCMEC/D3 monolayer and the co-culture incubated for 90 min. Non-adherent cells were washed with pre-warmed PBS and the relative number of adherent cancer cells was evaluated using a POLARstar™ OPTIMA microplate reader (BMG Labtech, Aylesbury, UK). All experiments were conducted independently three times in triplicate.

#### 4.7.2. Qualitative Adhesion Assay

hCMEC/D3 cells were seeded on fibronectin coated glass coverslips under the same conditions that were used in the CytoSelect™ adhesion assay. NSCLC cells were tagged with green fluorescent dye, seeded onto an activated hCMEC/D3 monolayer and incubated for 90 min. Non-adherent cancer cells were washed off and cells were fixed with 4% PFA. Semi-quantification of adhesion was assessed using confocal images and Zeiss ZEN software (Zeiss LSM 510 Meta Axioskop2, Carl Zeiss, Cambridge, UK).

### 4.8. Viability Assays

To determine the effect of blocking with CD15s-monclonal antibody on cell viability, NSCLC cells were seeded at 1 × 10^4^ in 96-well plates and incubated in growth medium supplemented with CD15s monoclonal antibody at a dilution of 1:100. Cell viability was then measured at different time points using CellTiter 96^®^ Aqueous one solution cell proliferation assay (MTS) (Promega UK, Southampton, UK). Absorbance was measured at 490 nm using a microplate reader (BMG Labtech, Aylesbury, UK).

### 4.9. Cancer Cell Adhesion under Shear Stress and Live Cell Imaging Microscopy

To assess the adhesion of cancer cell–brain endothelium under shear stress, a Vena8 Endothelial™ biochip (channel volume: 2.69 µL) (Cellix Ltd, Dublin, Ireland) was used. The biochips were coated with a 0.5 mm layer of 10 µg/mL fibronectin solution, incubated for 1 h and washed with PBS in a humidified environment. Then, 1.5 × 10^6^ of hCMEC/D3 cells were seeded in each channel and left to adhere for 2 h. The biochips were then plugged in to a microfluidic pump (Cellix Ltd., Dublin, Ireland), set up on perfusion mode with a 10 mL/h and 2.5 dyn/cm^2^ volumetric rate and kept in an incubator at 37 °C, 5% CO_2_. The adhesion assay was conducted after 24–48 h and TNF-α (25 pg/mL) was added to the circulating medium. The biochip was connected to a Zeiss Axiovert 200M inverted live cell (time lapse) microscope at 37 °C, 5% CO_2_ and 1 × 10^6^ cells/mL green fluorescently tagged NSCLC cells were perfused onto the hCMEC/D3 monolayer at 2.5 dyn/cm^2^. The flow rate was controlled via a Mirus Evo nanopump (Cellix Ltd., Dublin, Ireland) and Vena Flux Assay software. Live cell images were taken once every 10 min over 2 h to monitor cancer cell adhesion on the brain endothelial cell monolayer. Volocity software (V5.4, PerkinElmer, Waltham, MA, USA) was used to generate movie sequences. The experiment was repeated three independent times in triplicate.

### 4.10. Immunohistochemistry (IHC)

IHC was conducted on paraffin embedded, formalin fixed tissue sections (FFPE) of human lung to brain metastatic cancer biopsies using antibodies to CD15s and CD62E. Briefly, 4 μm thick FFPE sections were dewaxed in Xylene, rehydrated in 100%, 95%, 70% ethanol and deionized water respectively followed by 40 min of heat-induced epitope retrieval with citrate buffer (6.0 pH) at 95 °C and incubated with 3% hydrogen peroxide in methanol for 30 min to block endogenous peroxidase and biotin activity. Tissues were then blocked with 3% horse serum for 30 min and probed with primary antibodies for 1 h at room temperature. The Elite Vector Stain ABC system (Vector Labs, Burlingame, CA, USA) was employed as a detection system and DAB stain (Vector Labs, Burlingame, CA, USA) was used as a chromogen. Meyer’s haematoxylin (Sigma-Aldrich, Gillingham, UK) was used as a counterstain. Mouse IgM and IgG isotopes were used instead of primary antibodies in negative controls. Tissue sections were examined using a bright field automated microscope Ariol (Leica) for qualitative image analysis.

### 4.11. Confocal Microscopy

ICC images were obtained using the X63 oil immersion objective of a Zeiss LSM 510 Meta Axioskop 2 confocal microscope using lasers with excitation wave lengths of 405 nm (blue), 488 nm (green), 568 nm (red) and 674 nm (purple), with diode, argon and HeNe1 lasers, respectively. Identical settings were used to image negative controls in which primary antibody was used by non-specific Isotype.

### 4.12. Statistical Analysis

All experiments were conducted independently three times and the data expressed as ±SE. One-way ANOVA followed by Tukey’s multiple comparison post-hoc tests using Graph Pad Prism 6 software (version 6.1, UK) was used for statistical analysis.

## Supplementary Materials

Supplementary materials can be found at www.mdpi.com/1422-0067/18/7/1474/s1.
